# Serotonergic hallucinogens/psychedelics could be promising treatments for depressive and anxiety disorders in end-stage cancer

**DOI:** 10.1186/s12888-019-2288-z

**Published:** 2019-10-28

**Authors:** Rafael Guimarães dos Santos, José Carlos Bouso, Jaime E. C. Hallak

**Affiliations:** 1 Departamento de Neurociências e Ciências do Comportamento, Faculdade de Medicina de Ribeirão Preto, Universidade de São Paulo (USP), Hospital das Clínicas, Terceiro Andar, Av. Bandeirantes, Ribeirão Preto, São Paulo, 3900 Brazil; 2 Instituto Nacional de Ciência e Tecnologia – Medicina Translacional, Ribeirão Preto, Brazil; 3 ICEERS Foundation (International Center for Ethnobotanical Education, Research and Services), Barcelona, Spain

**Keywords:** Psychiatry, Psycho-oncology, End-stage cancer care, Depression, Anxiety, Hallucinogens, Psychedelics, Lysergic acid diethylamide, Psilocybin

## Abstract

In a recent issue of the BMC Psychiatry, the evidence of effectiveness of treatments for psychiatric conditions in end-stage cancer patients was reviewed (Johnson, 2018). The review was comprehensive, and included traditional and non-traditional/alternative treatments, including herbal medicines and spirituality. However, evidence showing that classic or serotonergic hallucinogens/psychedelics such as psilocybin and lysergic acid diethylamide (LSD) could be effective treatments for depressive and anxiety disorders in end-stage cancer was not included. In this commentary, we expand the information available on the original article by briefly reviewing data from recent placebo-controlled, double-blind, cross-over clinical trials showing evidence that administration of single (or few) doses of LSD and psilocybin was associated with rapid and sustained reductions in depressive and anxiety symptoms in patients with end-stage cancer and other life-threatening diseases (e.g., Bechterew’s disease, Parkinson’s disease, Celiac disease). Since these substances seem to produce rapid and sustained therapeutic effects with single (or few) doses and well tolerated, large-scale, prospective, multi-site studies of end-stage cancer and classical/serotonergic hallucinogens/psychedelics should be performed to improve our understanding of the therapeutic potentials of these drugs and their use on clinical practice.

In a recent review article published on BMC Psychiatry, the evidence of effectiveness of treatments for psychiatric conditions in end-stage cancer patients was critically and rigorously assessed [1]. The review is a comprehensive evaluation of the state of research in this area, regarding specifically the treatment for delirium, depression and anxiety. The author performed a complex, five-phase systematic strategy to find citations using four databases (PubMed, Medscape, Cochranes, and Oxford), and included “abstracts, reviews, raw reports, observational studies, and (random) controlled clinical trials found relevant”. Importantly, the review included not only conventional pharmacological treatments, but also non-traditional/alternative therapies and spirituality intervention, unlike previous reviews. Therefore, it is a timely and welcome review on an important topic.

However, we observed that the review did not include studies on classical or serotonergic hallucinogens/psychedelics such as lysergic acid diethylamide (LSD) and psilocybin in end-stage cancer. This is somewhat surprising, since these compounds were investigated for treating symptoms of depression and anxiety in patients suffering life-threatening diseases since the early 1960’s [2]. A recent systematic review showed that 11 clinical trials, involving a total of 445 participants, were performed between 1964 and 2016 assessing the effects of serotonergic hallucinogens/psychedelics in this clinical population [2]. Five open-label studies, using LSD or dipropyltryptamine (DPT) (another serotonergic hallucinogen/psychedelic), were published between 1960 and 2000. Four randomized, double-blind, placebo-controlled, cross-over studies, using LSD or psilocybin, were published between 2000 and 2017. The review concluded that “Evidence supports that patients with life threatening diseases associated with symptoms of depression and anxiety benefit from the anxiolytic and antidepressant properties of serotonergic hallucinogens” [2]. Moreover, some studies reported improved quality of life and reduced fear of death in patients, and treatments were well tolerated and associated with low rates of adverse effects.

Since the four more recent trials reviewed in the above-mentioned systematic review (2011–2016) are all randomized, double-blind, placebo-controlled, cross-over clinical trials, only their data will be briefly discussed further [3–7]. In the first of these cross-over trials, performed in the United States, a single dose of psilocybin (0.2 mg/kg) or an active placebo (niacin, 250 mg), associated with psychological support, were administered for 12 patients with anxiety and depressive disorders associated with advanced-staged cancer [3]. Compared to niacin, psilocybin administration was associated with significant reductions in anxiety symptoms (State-Trait Anxiety Inventory, trait anxiety) one and 3 months after treatment, and in depressive symptoms (Beck Depression Inventory) 6 months after treatment. Psilocybin was well tolerated, inducing only mild and transitory statistically significant elevations of heart rate and blood pressure when compared with niacin placebo.

Score values, level of significance, statistical tests, and questionnaires used in each study are reported on Table 1.

**Table 1:** Score values, level of significance, and questionnaires used in all cross-over studies analyzed

Reference	Questionnaires	Values, Level of significance, and Statistical tests
#3	STAI (trait anxiety) BDI	-STAI^a^: *1-month follow-up*: baseline: 43; 1 month: 36; *t*_11_ = 4.36, *P* = .001; *3-month follow-up*: 3 months: 37; *t*_10_ = 2.55, *P* = .03 -BDI^a^: *6-month follow-up*: baseline: 16; 6 months: 7; *t*_*7*_ = 2.71, *P* = .03 -*t* tests
#4	STAI (state anxiety)	−*2-month follow-up*: baseline: mean (SD) 53.1 (4.7); 2 months: 41.5 (3.2); *P* = .021 (*F* = 4.846, *df* = 2.18 -Repeated-measures analysis of variance (ANOVA) corrected for multiplicity
#6	BDI BSI GRID HADS HAM-A POMS STAI	−*5-week follow-up* -BDI - low dose: baseline: 18.40 (1.09), 5 weeks: 12.92 (1.58); high dose: baseline: 17.77 (1.61), 5 weeks: 7.00 (1.39); *P* < .05; difference between doses: *P* < .01 -BSI - low dose: baseline: 41.76 (4.40), 5 weeks: 33.74 (4.47); high dose: baseline: 40.19 (3.71), 5 weeks: 18.08 (3.62); *P* < .05; difference between doses: *P* < .01 -GRID - low dose: baseline (mean (SD)): 22.32 (0.88), 5 weeks: 14.80 (1.45); high dose: baseline: 22.84 (0.97), 5 weeks: 6.64 (1.04); *P* < .05; difference between doses: *P* < .001 -HADS - low dose: baseline: 9.48 (0.71), 5 weeks: 6.04 (0.79); high dose: baseline: 9.81 (0.69), 5 weeks: 3.92 (0.74); *P* < .05; difference between doses: *P* < .05 -HAM-A - low dose: baseline: 25.68 (0.89), 5 weeks: 16.64 (1.53); high dose: baseline: 25.73 (1.11), 5 weeks: 8.48 (1.16); *P* < .05; difference between doses: *P* < .001 -POMS - significant difference observed only with the high dose (baseline: 56.93 (5.33), 5 weeks: 18.96 (5.78); *P* < .05) and among doses (*P* < .01) -STAI - low dose: baseline: 47.46 (1.62), 5 weeks: 40.48 (2.11); high dose: baseline: 47.73 (1.91), 5 weeks: 34.64 (1.84); *P* < .05; difference between doses: *P* < .05 − *6-month follow-up*^b^: -BDI: 8.00 (1.50), 6.17 (1.26); *P* < 0.001 -BSI: 4.64 (0.72), 3.46 (0.66); *P* < 0.001 -GRID: 6.95 (1.24), 6.23 (1.30); *P* < 0.001 -HADS: 4.64 (0.72), 3.46 (0.66); *P* < 0.001 -HAM-A: 7.95 (1.19), 7.04 (1.17); *P* < 0.001 -POMS: 23.50 (6.57), 12.52 (5.36); *P* < 0.001 STAI: 36.83 (2.08), 35.32 (2.18); *P* < 0.001 *-z*-tests
#7	BDI HADS STAI	-BDI: *P*⩽.01 (1 day, 2 and 6 weeks), *P* < .05 (7 and 26 weeks) -HADS Anxiety: *P* < .05 (1 day and 26 weeks), *P*⩽.01 (2, 6, and 7 weeks) -HADS Depression: *P*⩽.001 (1 day and 6 weeks), *P* < .01 (2 and 7 weeks), *P* < .05 (26 weeks) -HADS Total: *P*⩽.001 (1 day, 2, 6 and 7 weeks), *P* < .05 (26 weeks) -STAI State: *P*⩽.01 (1 day and 7 weeks), *P*⩽.001 (2 and 6 weeks), *P* < .05 (26 weeks) -STAI Trait: *P*⩽.01 (1 day), *P*⩽.001 (2, 6 and 7 weeks), *P* < .05 (26 weeks) -*t*-tests (*P* values for 1 day and 2, 6, and 7 weeks pre-crossover, and for 26 weeks post-crossover)

*BDI* Beck Depression Inventory, *BSI* Brief Symptom Inventory, *GRID* GRID-Hamilton Depression Rating Scale, *HADS* Hospital Anxiety and Depression Scale, *HAM-A* Hamilton Anxiety Rating Scale, *POMS* Profile of Mood States, *STAI* State-Trait Anxiety Inventory

^a^These values are approximate, based on visual inspection of the graphics, since the values are not given in the original text

^b^Values represent mean (SD) for low-dose-1st/high-dose-2nd versus high-dose-1st/low-dose-2nd)

In the second cross-over study, performed in Switzerland, 12 patients with anxiety and depression associated with life-threatening diseases (e.g., cancer, Bechterew’s disease, Parkinson’s disease, Celiac disease) received psychological support associated with a single dose of 200 (experimental dose, *n* = 8) or 20 (active placebo, *n* = 4) μg of LSD [4, 5]. Compared with the active placebo, the higher dose of LSD was associated with significant reductions in anxiety symptoms (State-Trait Anxiety Inventory, state anxiety) 2 months after treatment (Table 1). Some patients also reported “facilitated access to emotions, enhanced introspective abilities, sustained reductions in anxiety and fear of death, and improved quality of life and physical well-being”. LSD was well tolerated, since it did not produce any severe adverse events (medical or psychiatric emergencies requiring hospitalization). The most common (> 20% prevalence) adverse reactions were anxiety, emotional distress, cold, feeling abnormal, gait disturbance, and sensorial illusions. LSD did not significantly alter blood pressure or heart rate. The experimental dose induced more adverse reactions than the active placebo.

In a more recent cross-over study performed in the United States, 51 patients suffering anxiety and depression associated with life-threatening cancer received psychological support and a high (22 or 30 mg/70 kg: 30 mg *n* = 1, 22 mg *n* = 29) or low (1 or 3 mg/70 kg: 1 mg *n* = 38; 3 mg *n* = 12) psilocybin dose [6]. Compared to the low psilocybin dose, high-dose psilocybin was associated with significant reductions in clinician- and self-rated measures of depression and anxiety (Hamilton Depression Rating Scale, Hamilton Anxiety Rating Scale, Beck Depression Inventory, State-Trait Anxiety Inventory, Hospital Anxiety and Depression Scale, Brief Symptom Inventory, Profile of Mood States). Moreover, the difference between baseline and the 6 months follow-up across groups was significant for all measures (see detailed results in Table 1).

As in the other trials, patients reported “increases in measures of quality of life, life meaning, death acceptance, and optimism”. These effects were corroborated by friends, family, or work colleagues. Moreover, psilocybin was well tolerated, and no serious adverse events occurred. Transient and moderate increases in blood pressure and heart rate were observed after psilocybin. Nausea and vomiting were observed in 15% of participants but only with the higher dose. The higher psilocybin dose induce more adverse reaction than the lower dose, incuding physical (21% versus 8%) and psychological discomfort (32% versus 12%), anxiety (26% versus 15%) and headache (three participants in the high-dose group). One volunteer in the high-dose group experienced transient paranoid ideation, but no cases of hallucinogen persisting perception disorder or prolonged psychosis were observed.

The last cross-over study was also performed in the United States and included 29 patients with anxiety and depression associated with life-threatening cancer. Psychological support with a high dose of psilocybin (0.3 mg/kg) was compared to psychological support and niacin (active placebo, 250 mg) [7]. Compared to niacin, high-dose psilocybin was associated with significant reductions in depression and anxiety symptoms 1 day and 2, 6, 7, and 26 weeks post-dosing (Beck Depression Inventory, State-Trait Anxiety Inventory, Hospital Anxiety and Depression Scale) (Table 1).

Participants also reported “decreases in hopelessness, improved spiritual wellbeing, and increased quality of life”. Psilocybin was well tolerated, inducing mild and transitory elevations on blood pressure and heart rate. There were no serious adverse reactions. The most common adverse reactions were headaches/migraines (28%), nausea (14%), transient anxiety (17%), and transient psychotic-like symptoms (7%: one case of transient paranoid ideation and one case of transient thought disorder).

Despite the small sample sizes of the two first trials and the cross-over design of all studies, results consistently showed that single or few doses of serotonergic hallucinogens/psychedelics can be effective for reducing depression and anxiety symptoms in patients with end-stage cancer. Importantly, in all studies reviewed above, these compounds were well tolerated and produced rapid and sustained improvements in anxiety and depression [2]. Moreover, these drugs induced improvements in spiritual well-being and quality of life, and less fear of death among these patients. Furthermore, in the two most recent studies [6, 7], psilocybin administration was associated with mystic-like experiences, which seem to mediate the therapeutic effect of these drugs on anxiety and depression, since changes in this measure correlated with changes in the scores of most primary outcome measures of anxiety and depression on both studies (GRID-HAMD, HAM-A, HADS, BDI, STAI, POMS, BSI) [2, 6, 7]. This is especially relevant considering that the review by Johnson reported that “Recently, small yet promising trials testing the effectiveness of interventions designed to boost spirituality have found that spirituality can positively affect end-stage cancer patients’ mood and quality of life” ([1], pg. 9).

Evidence that serotonergic hallucinogens/psychedelics have antidepressive and anxiolytic properties is also available from other human studies. Recent evidence from open-label [8] and controlled studies [9, 10] showed that administration of single doses of the Amazonian botanical decoction ayahuasca (rich in the hallucinogenic/psychedelic tryptamine dimethyltryptamine or DMT) is associated with antidepressive and anxiolytic effects in healthy volunteers [9] and in patients with treatment-resistant major depressive disorder (MDD) [8, 10]. A recent open-label study of single-dose psilocybin for patients with treatment-resistant MDD also showed positive results [11].

Thus, considering the evidence of effectiveness presented above, we suggest that classical or serotonergic hallucinogens/psychedelics should be included as some of the pharmacological treatment options for depression and anxiety in end-stage cancer, especially if we consider that the treatment of depression in end-stage cancer patients “is based heavily on expert opinion extrapolated from case reports and/or small non-end-stage cancer patient studies” ([2], pg. 6). Moreover, these studies report a new form of intervention that includes psychotherapy associated with drug therapy to stimulate mystic-like experiences, which seem to be one of the mechanisms behind the therapeutic effects of these drugs [3, 6, 7]. More importantly, these substances seem to produce rapid and sustained therapeutic effects with single or few doses and are associated with few adverse reactions, which is the opposite from traditional pharmacological therapies, which are often taken in a daily basis, for prolonged periods of time, and are associated with several adverse reactions. Large-scale, prospective, multi-site studies of end-stage cancer and classical or serotonergic hallucinogens/psychedelics should be performed to improve our understanding of the therapeutic potentials of these drugs and their use on clinical practice.

## Data Availability

Not applicable.

## Abbreviations

DMT: Dimethyltryptamine
DPT: Dipropyltryptamine
LSD: Lysergic acid diethylamide
MDD: Major depressive disorder
